# Supplementary material for: Punctuated evolution of canonical genomic aberrations in uveal melanoma
Source: Nat Commun. 2018 Jan 9;9:116. doi: 10.1038/s41467-017-02428-w (PMC5760704; doi:10.1038/s41467-017-02428-w)
Supplement: Supplementary file 1 — Supplementary Information [file 41467_2017_2428_MOESM1_ESM.pdf]

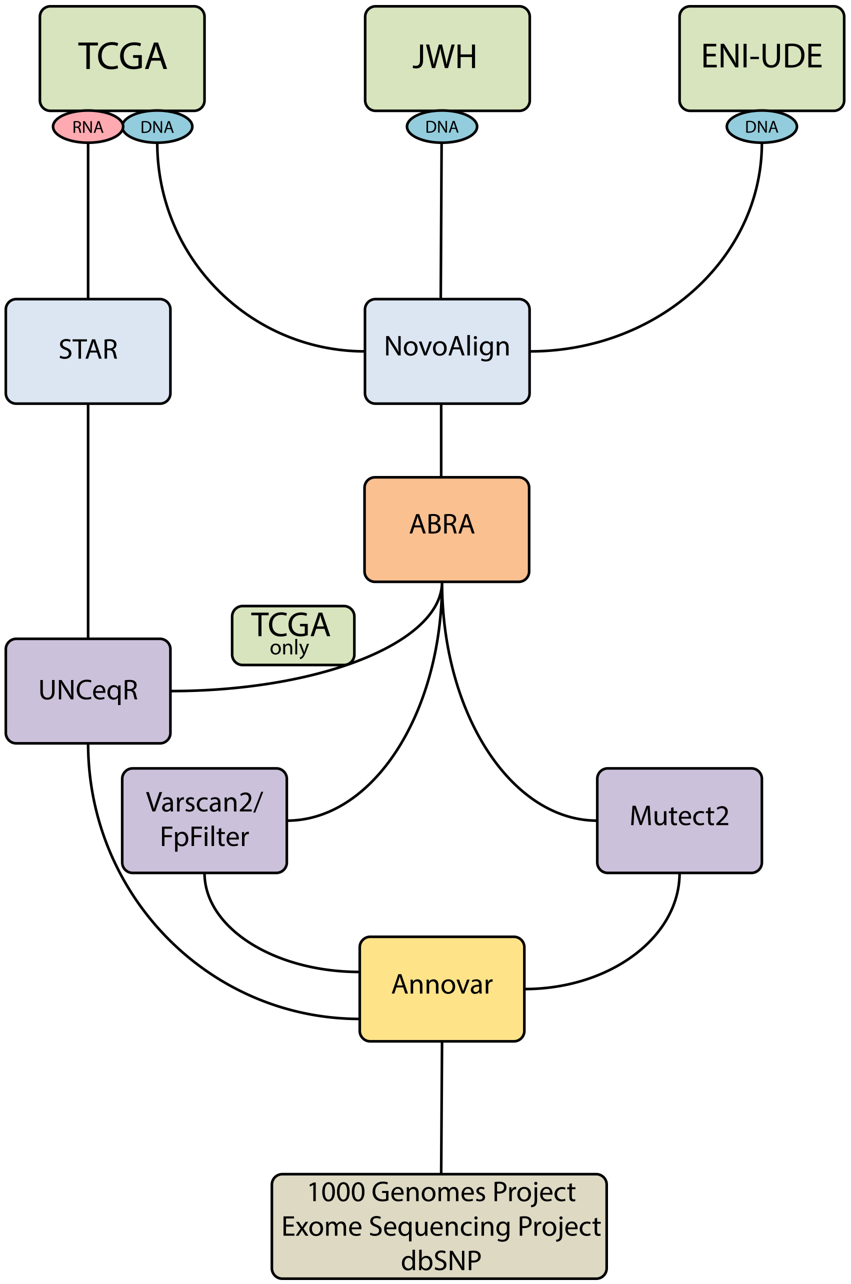

Supplementary Figure 1. Enhanced pipeline for automated detection of somatic mutations from whole exome sequencing (WES) data from primary uveal melanomas. Matched WES from three different sources (green boxes) underwent alignment with Novoalign (blue boxes), indel realignment with ABRA (orange boxes) and mutation calling with Varscan2 and MuTect2 (purple boxes). RNA-Sequencing from TCGA was aligned with STAR (blue boxes) and was used in combination with TCGA WES data to rescue mutations in low coverage exome regions using UNCeqR (purple boxes). Somatic mutations were annotated using Annovar (yellow boxes) and filtered based on population genetics using 1000 Genomes Project, Exome Sequencing Project, and dbSNP (tan boxes). Additional techniques used to detect mutations are described in Figure 2 and the methods section, including strategies to detect “SpliceDels” and deep deletions.

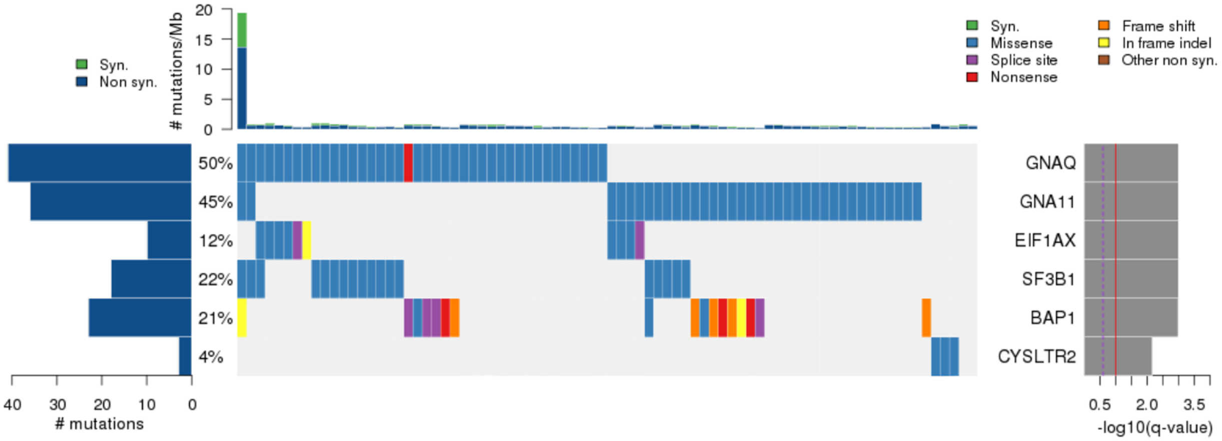

Significantly Mutated Genes

Column Descriptions:

- nnon = number of (nonsilent) mutations in this gene across the individual set
- npat = number of patients (individuals) with at least one nonsilent mutation
- nsite = number of unique sites having a non-silent mutation
- nsil = number of silent mutations in this gene across the individual set
- p = p-value (overall)
- q = q-value, False Discovery Rate (Benjamini-Hochberg procedure)

Table 1. A Ranked List of Significantly Mutated Genes. Number of significant genes found: 6.

rank	gene	longname	codelen	nnei	nncd	nsil	nmis	nstp	nspl	nind	nnon	npat	nsite	pCV	pCL	pFN	p	q
1	GNAQ 1	guanine nucleotide binding protein (G protein), q polypeptide	1106	1000	0	0	40	1	0	0	41	40	4	1e-16	1e-05	0.0014	1e-16	6.1e-13
2	GNA11 2	guanine nucleotide binding protein (G protein), alpha 11 (Gq class)	1104	354	0	0	36	0	0	0	36	36	3	2.6e-16	1e-05	0.0028	1e-16	6.1e-13
3	EIF1AX 3	eukaryotic translation initiation factor 1A, X-linked	459	174	0	0	7	0	2	1	10	10	6	1e-16	1e-05	0.01	1e-16	6.1e-13
4	SF3B1 4	splicing factor 3b, subunit 1, 155kDa	4035	20	0	0	18	0	0	0	18	18	5	1.1e-11	1e-05	0.92	4e-15	1.8e-11
5	BAP1 5	BRCA1 associated protein-1 (ubiquitin carboxy-terminal hydrolase)	2254	1	4	0	4	3	0	16	23	17	23	5.5e-10	0.43	0.67	7.1e-09	0.000026
6	CYSLTR2 6	cysteinyl leukotriene receptor 2	1043	1000	0	0	3	0	0	0	3	3	1	0.00011	0.001	0.0047	1.9e-06	0.0059

Supplementary Figure 2. Significantly enriched mutations detected in the uveal melanoma TCGA dataset using the GDAC Firehose Pipeline[1].

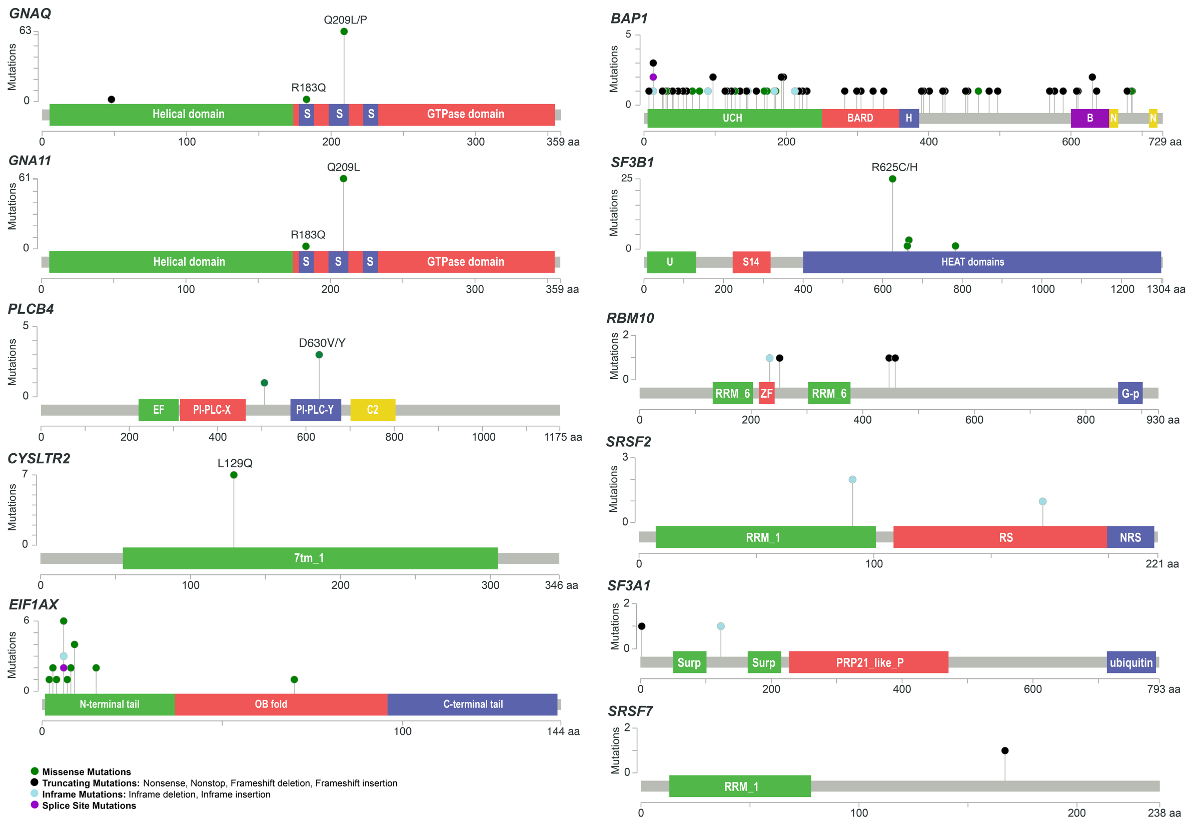

Supplementary Figure 3. Lollipop plot showing distribution of mutations in *GNAQ*, *GNA11*, *PLCB4*, *CYSLTR2*, *BAP1*, *SF3B1*, *EIF1AX*, and the newly discovered splicing genes (*RBM10*, *SRSF2*, *SF3A1*, *SRSF7*) in primary UMs. S, Switch regions; EF, Phosphoinositide-specific phospholipase C domain; PI-PLC-X, Phosphatidylinositol-specific phospholipase C, X domain; PI-PLC-Y, Phosphatidylinositol-specific phospholipase C-Y domain; C2, C2 domain; 7tm_1, 7 transmembrane receptor (rhodopsin family) domain; UCH, ubiquitin carboxyl-terminal hydrolase catalytic domain; BARD, BRCA1-associated RING domain; H, HCF1 binding motif; B, BRCA1 binding domain; N, nuclear localization sequences; U, U2AF2 interaction motif; S14, SF3B14 interaction motif; HEAT, Huntingtin, elongation factor 3, protein phosphatase 2A, and TOR1 domains; OB fold, oligonucleotide binding fold. RRM_6, RNA recognition motif; ZF, Zn-finger in Ran binding protein domain; G-p, G-patch domain; RRM_1, RNA recognition motif; RRM_1 pfama, RNA recognition motif; RS, Arginine/Serine rich domain; NRS, Nuclear retention signal; Surp, Surp module; PRP21_like_P, Pre-mRNA splicing factor PRP21 like protein domain. Plots were generated using the cBioPortal visualization tool MutationMapper[2], and domain information was populated based on default annotations in combination with a review of the literature.

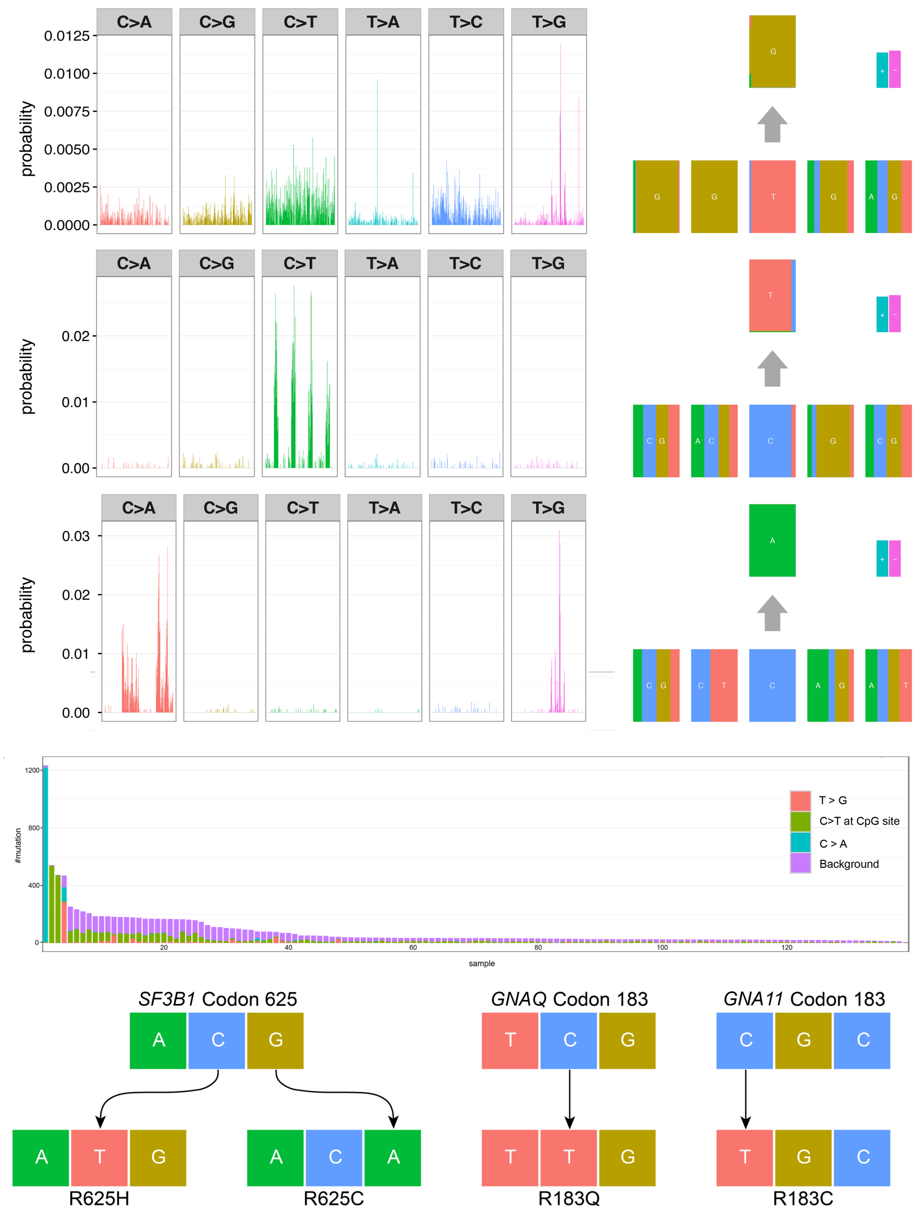

Supplementary Figure 4. Mutational signature analysis of uveal melanoma. SNPs from all samples with WES data from tumor and matching blood (n=139) were extracted and subjected to a mutational signature analysis using the pmsignature package in R, which takes nucleotide base changes into account in combination with flanking bases. Three signatures were found. The most prevalent signature was a C>T mutation at CpG sites, which is an aging signature. Hotspot mutations in *GNAQ/11* at codon 183 and *SF3B1* at codon 625 are representative of this signature. The second most prevalent was a T>G transversion, which has an unknown cause. Third was a C>A transversion, which is associated with oxidative stress. Recommended parameter testing was followed where an incremental number of mutational signatures were modeled. The number and type of mutational signatures was selected at the point before addition of a new signature resulted in mutations being reassigned from an existing signature to a new one.

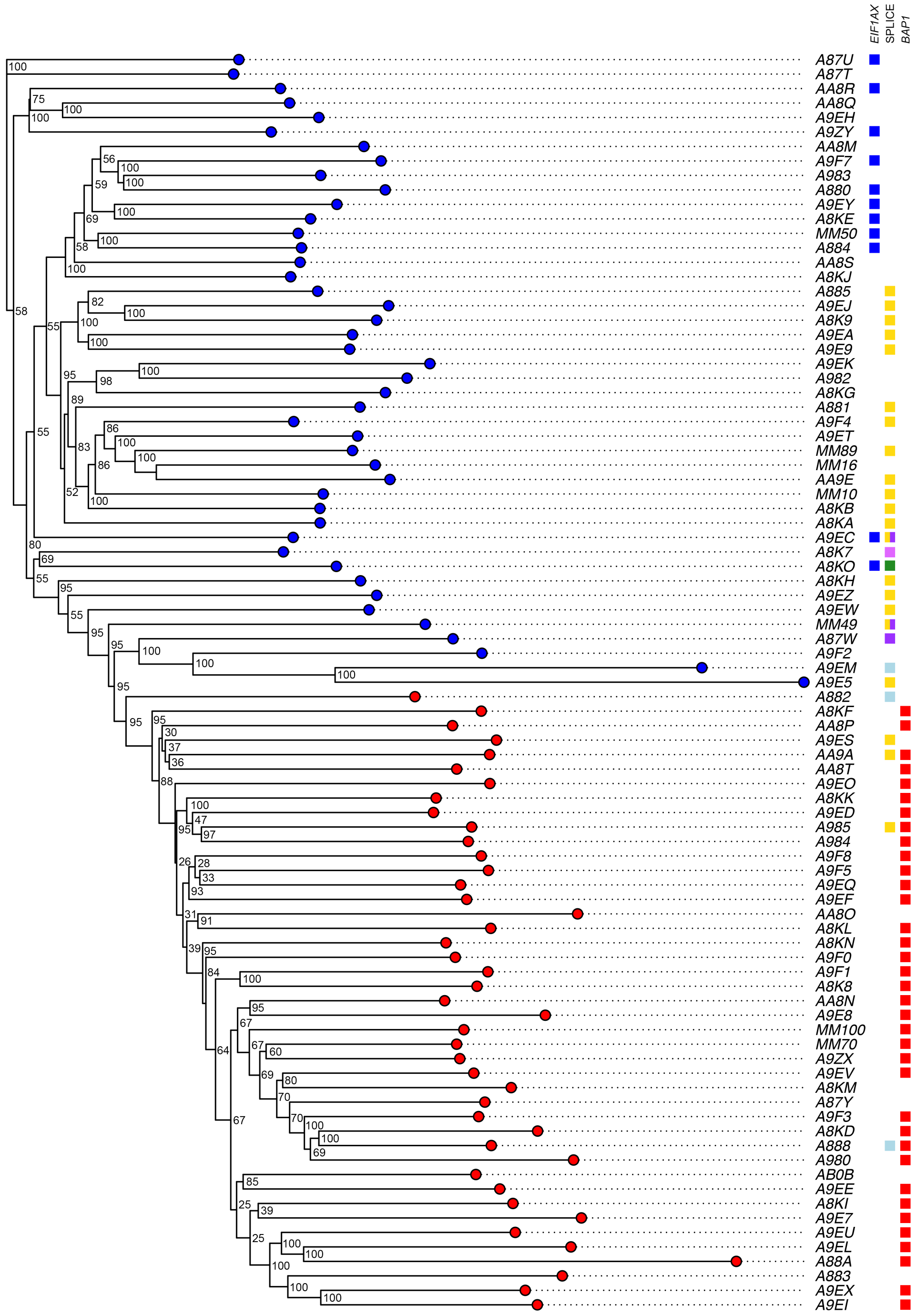

Supplementary Figure 5. Phylogenetic relationships between uveal melanoma samples using a minimum evolution algorithm with a Canberra distance matrix[35] and displayed with a rooted tree. A bootstrapping analysis of tree bipartitions (100 replicates) was conducted to estimate the percent likelihood for each partition. For example, a nodal value of 95 means that out of 100 reconstructed trees that specific partition occurred 95 out of 100 times.

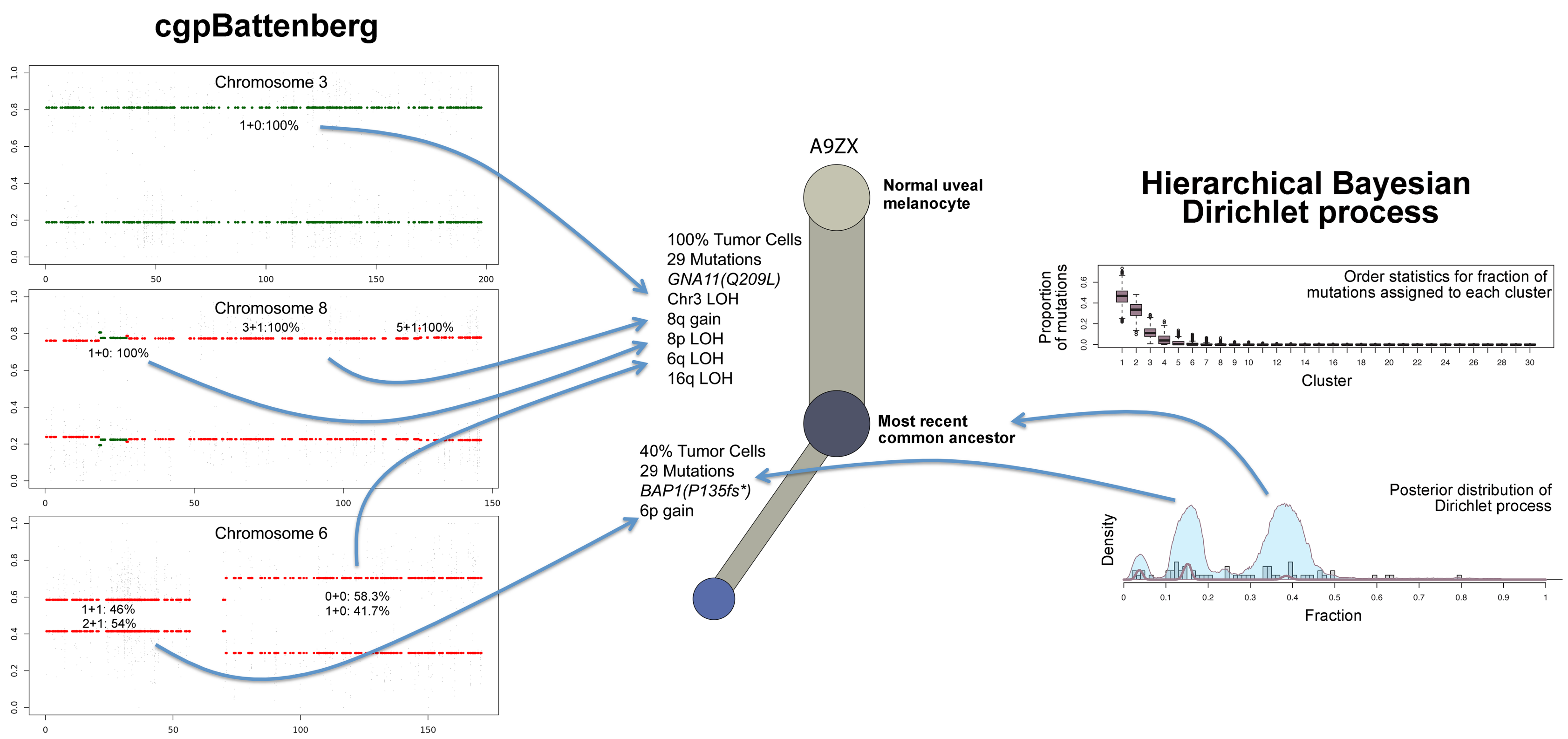

Supplementary Figure 6. Overview of clonality analysis methodology using cgpBattenberg to estimate clonal relationships between chromosome copy number alterations and a hierarchical Bayesian Dirichlet process for clonal relationships between mutations.

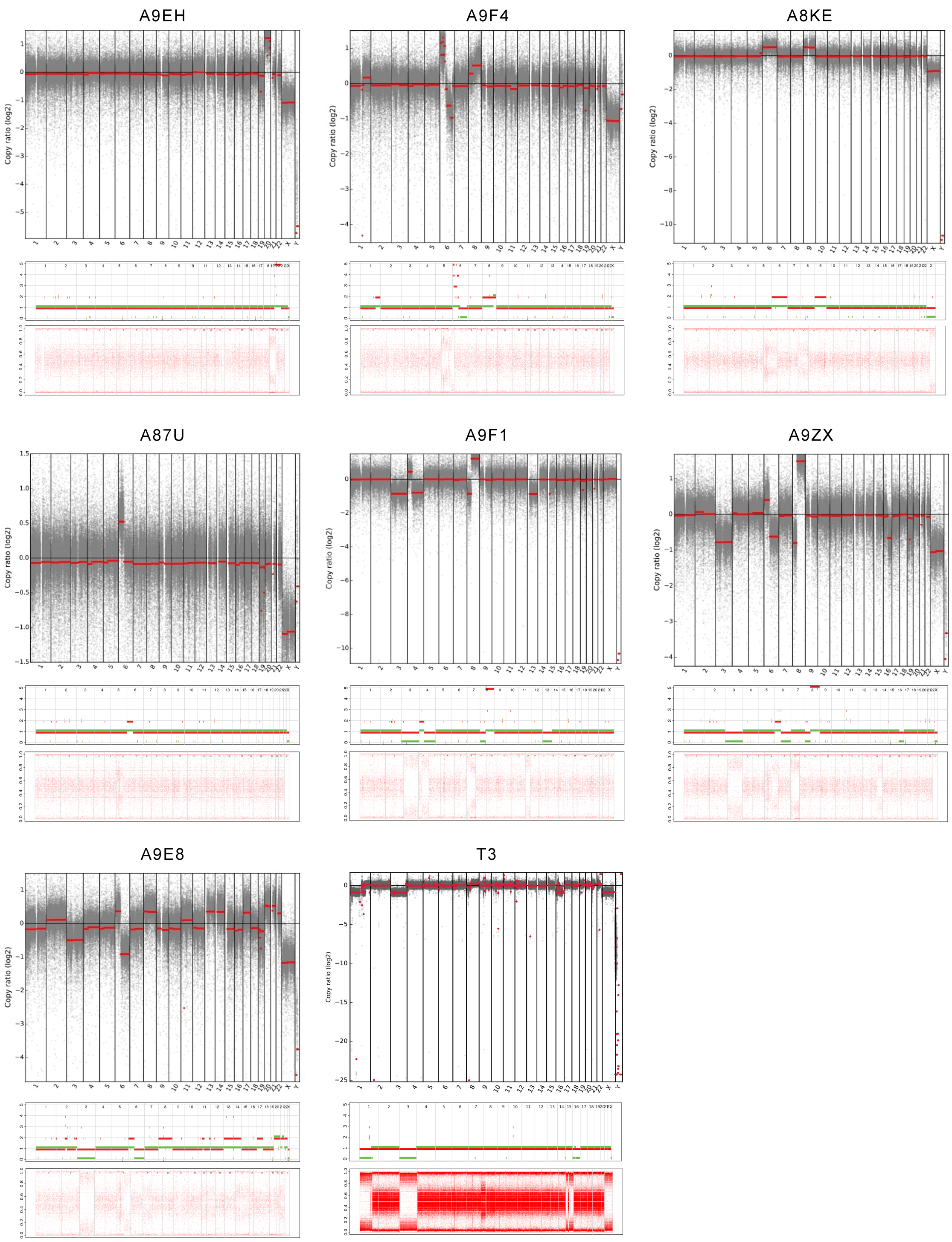

Supplementary Figure 7. Chromosome copy number aberration (CNA) plots from CNVkit (top panel) and ASCAT (middle panel) and minor allele frequency (MAF) plots (bottom panel) for the 8 life history clonal evolution cases.

Supplementary Table 1. Summary of clinical information for whole exome sequencing data

Variable	JWH (n=37)	TCGA (n=80)	UNI-UDE (n=22)
Age at diagnosis, y			
Mean	60.3	61.7	61.7
Median (Q1-Q3)	63.0 (51.2-67.5)	61.5 (51.0-74.3)	69 (48.5-73.3)
Sex, No.(%)			
Female	17 (45.9)	35 (43.8)	10 (54.5)
Male	20 (54.1)	45 (56.3)	12 (45.5)
Ciliary body involvement, No. (%)			
Yes	28 (75.7)	24 (30.0)	11 (50.0)
No	8 (21.6)	56 (70.0)	11 (50.0)
Not Available	1 (2.7)	0 (0.0)	0 (0.0)
Pathologic cell type, No. (%)			
Spindle	7 (18.9)	30 (36.1)	17 (77.3)
Mixed/epithelioid	28 (78.4)	53 (63.9)	2 (9.1)
Not Available	1 (2.7)	0 (0.0)	3 (13.6)
LBD, mm			
Mean	18.0	16.9	14.2
Median (Q1-Q3)	18 (16.0-20.0)	17 (14.8-19.4)	13.3(11.8-15.9)
Tumor thickness, mm			
Mean	10.3	10.4	10.3
Median (Q1-Q3)	10.4 (8.8-12.5)	10.5 (8.5-12.0)	10.6 (8.8-12.6)
Metastasis, No. (%)			
Yes	19 (51.3)	12 (15.0)	4 (18.2)
No	18 (49.7)	65 (81.2)	0 (0.0)
Not Available	0 (0.0)	3 (3.8)	18 (81.8)
Melanoma-specific mortality, No. (%)			
Yes	13 (37.8)	11 (13.8)	4 (18.2)
No	23 (52.2)	68 (85.0)	18 (81.8)
Not Available	0 (0.0)	1 (1.2)	0 (0.0)
All-cause mortality, No (%)			
Yes	12 (40.5)	13 (16.2)	4 (18.2)
No	22 (59.5)	67 (83.8)	0 (0.0)
Not Available	0 (0.0)	0 (0.0)	18 (81.8)
Follow-up, mo			
Mean	37	15	Not Available
Median (Q1-Q3)	31.5 (10.8-40.3)	12.9 (2.4-23.4)	Not Available

Supplementary References

1. Broad Institute TCGA Genome Data Analysis Center (2016): Analysis Overview for Uveal Melanoma (Primary solid tumor cohort) - 28 January 2016. Broad Institute of MIT and Harvard. doi:10.7908/C1Q23ZRV.

2. Gao J. *et al*. Integrative analysis of complex cancer genomics and clinical profiles using the cBioPortal. *Sci Signal* **6,** pl1 (2013).
