# Supplementary material for: Punctuated evolution of canonical genomic aberrations in uveal melanoma
Source: Nat Commun. 2018 Jan 9;9:116. doi: 10.1038/s41467-017-02428-w (PMC5760704; doi:10.1038/s41467-017-02428-w)
Supplement: Supplementary file 3 — Description of Additional Supplementary Files [file 41467_2017_2428_MOESM3_ESM.pdf]

Description of Supplementary Files

File Name: Supplementary Data 1
Description: Summary and list of mutations identified in whole exome sequencing data from 139 primary uveal melanoma samples.

File Name: Supplementary Data 2
Description: Summary and list of mutations identified in whole genome sequencing data from 12 primary uveal melanomas.
